# Comparison of MR findings of acute traumatic peripheral nerve injury and acute compressive neuropathy in a rat model

**DOI:** 10.1371/journal.pone.0240911

**Published:** 2020-11-19

**Authors:** Bo Ra Kim, Dong-Ho Ha, Jong Kuk Kim, Young Hee Kim

**Affiliations:** 1 Department of Radiology, Dong-A University Medical Center, Busan, Republic of Korea; 2 Department of Neurology, Dong-A University Medical Center, Busan, Republic of Korea; 3 Peripheral Neuropathy Research Center (PNRC), Dong-A University College of Medicine, Busan, Republic of Korea; McLean Hospital, UNITED STATES

## Abstract

**Purpose:**

The treatment strategy is different for acute traumatic peripheral nerve injury and acute compressive neuropathy. This study aimed to compare magnetic resonance imaging (MRI) features of acute traumatic peripheral nerve injury and acute compressive neuropathy in a rat model.

**Materials and methods:**

Twenty female Sprague-Dawley rats were divided into two groups. In the crush injury group (n = 10), the unilateral sciatic nerve was crushed using forceps to represent acute traumatic peripheral nerve injury. In the compression injury group (n = 10), the unilateral sciatic nerve was ligated using silk to represent acute compressive neuropathy. The MRI of eight rats from each group were acquired on postoperative days 3 and 10. Fat-suppressed T2-weighted images were acquired. Changes in the injured nerve were divided into three grades. A Fisher’s exact test was used to compare the changes in the nerves of the two groups. Histological staining and a western blot analysis were performed on one rat in each group on day 3. Neurofilament, myelin basic protein (MBP), and p75^NTR^ staining were performed. Expression of neurofilament, MBP, p75^NTR^, and c-jun was evaluated by western blot analysis.

**Results:**

MR neurography revealed substantial nerve changes in the compression injury group compared with the crush injury group at two-time points (p = 0.001 on day 3, p = 0.026 on day 10). The histopathological analysis indicated the destruction of the axon and myelin, mainly at the injury site and the distal portion of the injury in the crush injury group. It was prominent in the proximal portion, the injury site, and the distal portion of the injury in the compression injury group. The degree of axonal and myelin destruction was more pronounced in the compression injury group than in the crush injury group.

**Conclusion:**

MR neurography showed prominent and long-segmental changes associated with the injured nerve in acute compressive neuropathy compared with acute traumatic peripheral nerve injury.

## Introduction

Magnetic resonance imaging (MRI) is a useful modality for the clinical diagnosis and surgical planning of peripheral nerve disorders [1–7]. The most important roles of MRI include detection of causative space-occupying lesions such as neoplasm, hematoma, and bony fragments in peripheral neuropathy and determination of the changes in denervated muscles that indirectly reflect nerve injury [2]. However, changes in the denervated muscle occur several days after nerve injury [1, 8–11], and these changes occur only in the skeletal muscles innervated by the motor nerves.

MR neurography (MRN) is an advanced imaging technique that directly reveals the peripheral nerve with high contrast and high resolution. The use of MRN for early and differential diagnosis of peripheral neuropathy has recently increased [1–3, 6, 11–14]. However, further studies analyzing the MRN features of different types of peripheral neuropathy are necessary for differential diagnosis.

The most common causes of peripheral nerve injury are trauma and entrapment [15]. In compressive neuropathy, axonal and myelin damage induced by sustained compression results in nerve infarction and fibrosis [15]. Therefore, early and differential diagnosis is important for compressive neuropathy to avoid catastrophic sequelae.

Conservative treatment is preferred in traumatic peripheral nerve injury. However, immediate removal of the space-occupying lesion is necessary in compressive neuropathy for a favorable prognosis. Thus, the differential diagnosis of traumatic peripheral nerve injury and compressive neuropathy is essential for patients with peripheral neuropathy. Several studies have reported varying MRN findings of traumatic nerve injury associated with different degrees of injury [16–18]. However, few studies have focused on MRN features for the differential diagnosis of traumatic nerve injury and compressive neuropathy.

In this study, we compared the MRI and MRN findings of acute traumatic peripheral nerve injury and acute compressive neuropathy in a rat model. We also investigated the histopathological changes, which may be associated with MRN findings.

## Materials and methods

### Animals

**Fig 1:**
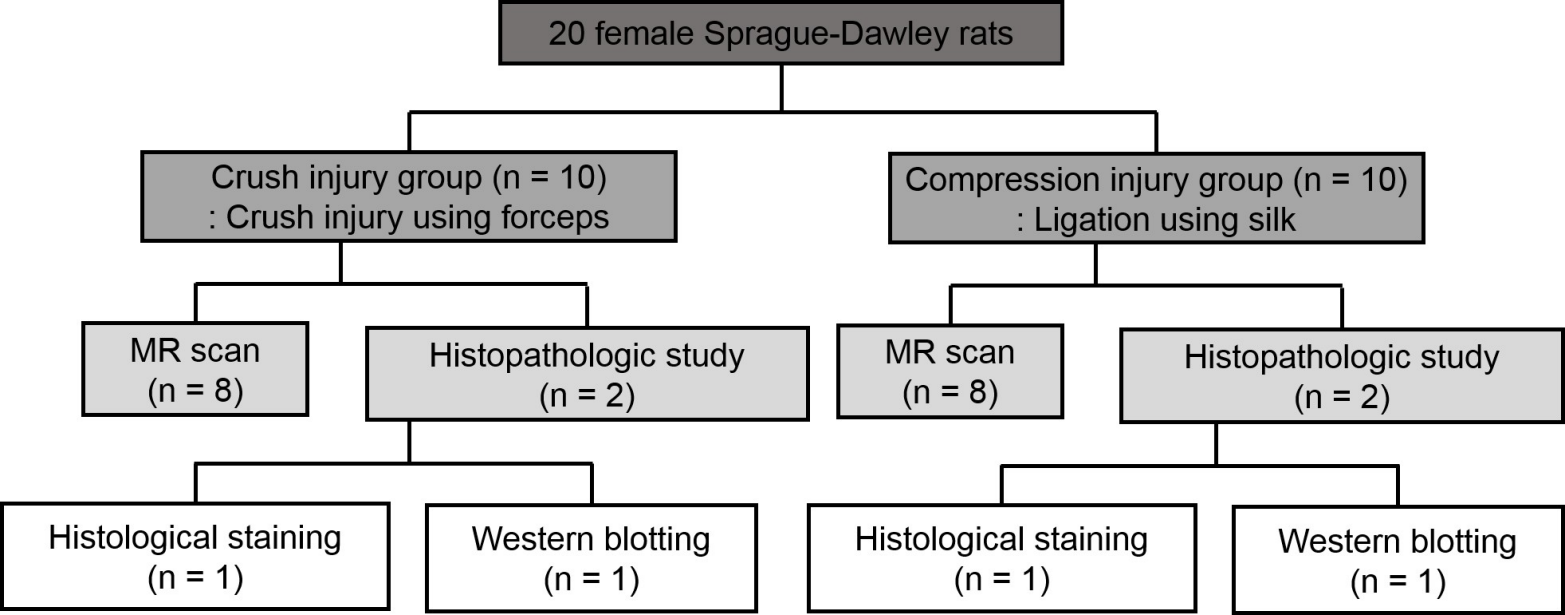
Flowchart of the study process in the two groups.

This study was approved by the Dong-A University Institutional Animal Care and Use Committee. Twenty female Sprague-Dawley rats aged seven weeks were divided into two groups: the crush injury group (n = 10) representing acute traumatic peripheral nerve injury, and compression injury group (n = 10) representing acute compressive neuropathy (Fig 1).

Twenty female rats, weighing 150–250 g, were obtained from Samtako Bio Korea Inc. (Osan, Korea) and were housed in a standard animal facility with a 12-hour diurnal cycle. All animals were acclimatized prior to surgery and allowed standard rat chow and water *ad libitum*.

After one week of acclimatization, the animals were fasted for 6 h without fluid restriction before anesthesia. The animals were anesthetized with 4–5% isoflurane using a vaporizer for surgery. When the rats showed no response to pinching at the hindlimb, one hindlimb of each rat was randomly selected for surgery, and the hair was removed using an electronic clipper. A 2-cm incision was made at the mid-thigh level, and the sciatic nerve was exposed at the sciatic notch. In the crush injury group, the sciatic nerve was crushed for 5 s using small forceps. In the compression injury group, the nerve was ligated using 4–0 silk, resulting in nerve compression. The procedures were conducted under sterile conditions, including sterilized surgical instruments and draping the incision site with betadine. In all cases, the surgical intervention took approximately 5–10 min, and the blood loss was minimal. After the surgery, the animals were individually placed in the cage and warmed up to 37°C until awakening from anesthesia. The rats showed limping gait immediately after the surgery and recovered with usual movement and ordinary food and water intake within 1–2 days. For that reason, the pain killer was not considered, and the rats showed normal weight gain of approximately 300–350 g when the MR scan or histopathological study was conducted.

### MR study

MR studies were conducted in 16 rats (eight rats per group) on days 3 and 10 after the surgery, with a 3 Tesla MR unit (Discovery MR 750; GE Healthcare, Milwaukee, WI, USA). Under general anesthesia using a mixture of 10 mg/kg xylazine hydrochloride (Rompun; Bayer, Leverkusen, Germany) and 10 mg/kg alfaxalone (Alfaxan; Jurox, NSW, Australia) injected intraperitoneally, the animals were placed in an eight-channel wrist array coil in prone position. The area from the lower abdomen to the middle portion of both lower legs was imaged.

Axial and sagittal fat-suppressed T2-weighted images were acquired, followed by oblique, coronal fat-suppressed T2-weighted images, which were set parallel to the course of the sciatic nerve [2, 19]. The scan parameters were as follows: repetition time = 3600 ms, echo time = 85 ms, matrix size = 320 × 192, field of view = 60 × 60 mm^2^, and slice thickness = 2 mm. Using the vendor-supplied software (Advantage Workstation; Version 4.3, GE Healthcare, Milwaukee, WI, USA), thin-slab maximum-intensity projection (MIP) reconstruction from the oblique coronal fat-suppressed T2-weighted images was obtained by one observer (with one year of experience in radiology).

MR findings of the compressed or crushed sciatic nerve and denervated muscle changes in the hindlimb were evaluated by two board-certified radiologists with 16 years and 1 year of experience in MRI interpretation, in a blinded manner and by consensus.

**Fig 2:**
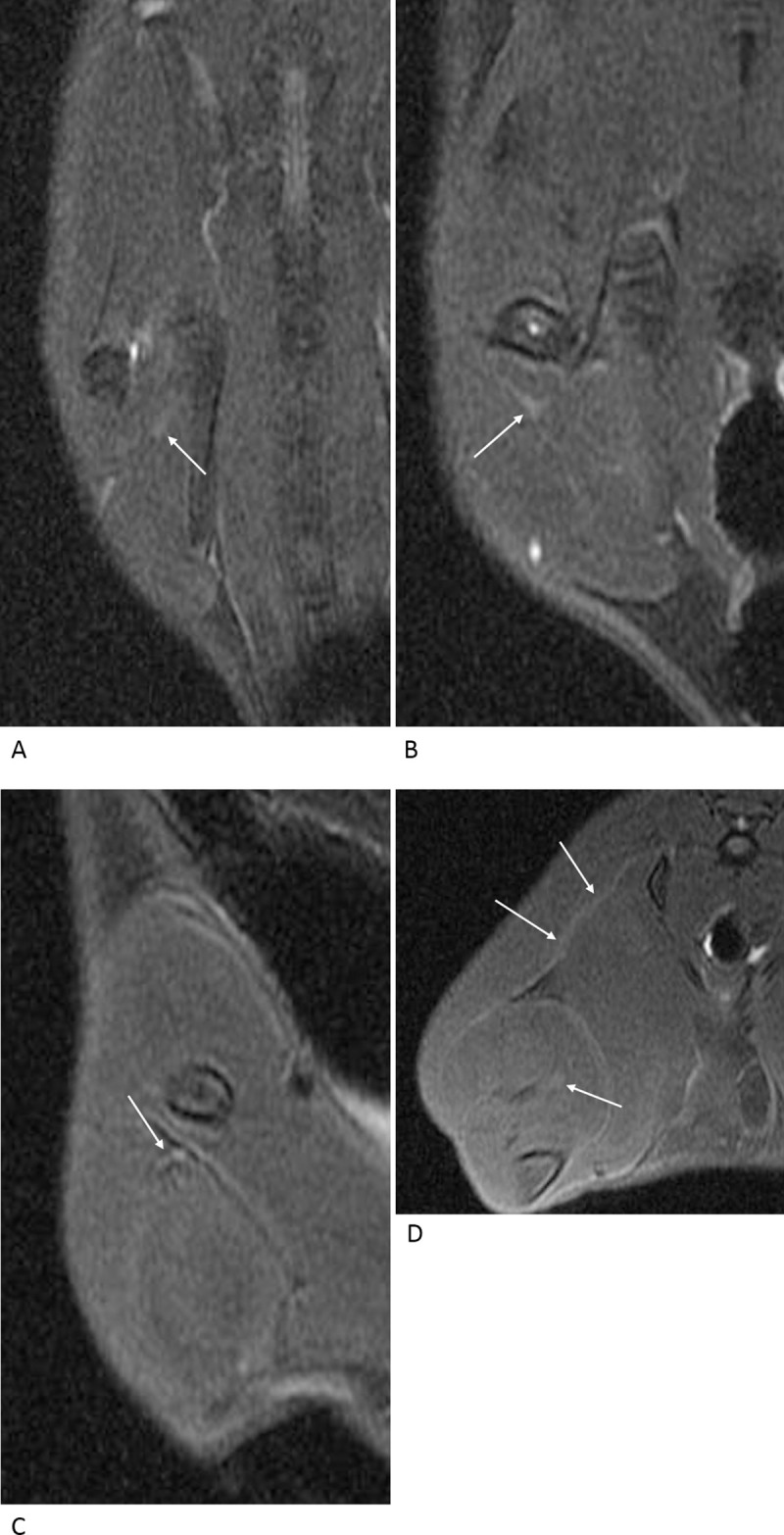
Normal nerve and muscle before surgical intervention. On the oblique coronal (A-C) and axial (D) fat-suppressed T2-weighted images, the expected locations of the left sciatic nerve are indicated by the arrows. However, the structures are not clearly distinguished from the intermuscular fascia. The calf muscles are isointense to the adjacent muscles.

Nerve changes were assessed according to axial and oblique coronal fat-suppressed T2-weighted images and MIP images using a three-point grading scale: grade 1, nerve not well visualized and cannot be distinguished from the normal nerve (Fig 2); grade 2, partially visualized nerve as a linear structure; and grade 3, thick and T2 hyperintense nerve. Concurrently, denervation changes in calf muscles were evaluated. Muscle changes were assessed based on axial and oblique coronal fat-suppressed T2-weighted images, and the visual changes in signal intensity were also classified into three grades: grade 1, iso-signal intensity compared to that of the adjacent muscles; grade 2, slight hyperintensity; and grade 3, distinct hyperintensity. To increase the sensitivity of lesion detection, the highest grade (both nerve and muscle changes) among the axial and oblique coronal fat-suppressed T2-weighted images and MIP images were selected.

After MRI, all animals were sacrificed by an overdose of CO_2_.

### Histopathologic study

A histological study of four rats was conducted on day 3 after the surgery. For histological staining, one rat in each group (two rats in total) was used. A western blot analysis was performed on the remaining two rats (one rat per group).

The animals were intracardially perfused with 150 mL of 0.05 mol/L phosphate-buffered saline, followed by 300 mL of fixative (4% paraformaldehyde, adjusted to pH 7.2). The injured nerves and the contralateral normal sciatic nerves were removed. For histological staining, the nerve tissue samples, measuring approximately 1 cm in length and including proximal and distal portions of the injury site, were obtained. For western blot analysis, the nerve tissue samples, approximately 0.5 cm in length, were collected immediately distal to the compression or crush site.

Histological staining revealed neurofilament/p75^NTR^ double staining and myelin basic protein (MBP)/p75^NTR^ double staining. The neurofilament is the supporting structure of the axon. MBP is a protein that plays an important role in peripheral nerve myelination. p75^NTR^ is a neurotrophic receptor that is increased in peripheral nerve injury. The stained sections were examined with a Zeiss Axio Imager 2 equipped with an ApoTome (Carl Zeiss, Göttingen, Germany) for light microscopic analysis.

The expression of neurofilament, MBP, p75^NTR^, and c-jun was analyzed via western blotting. Peripheral nerve injury rapidly increases the levels of c-jun, which is a transcription factor in Schwann cells. An enhanced chemiluminescence western blot system (Amersham, Piscataway, USA) was performed, followed by image analysis with LuminoGraph III (ATTO, Tokyo, Japan). The relative gray value of each target protein (gray value of a target band/gray value of GAPDH band) was calculated with a software CS analyzer (ATTO, Tokyo, Japan). At least three independent experiments were performed for the quantitative analysis.

### Statistical analysis

SPSS version 22 (IBM Corp., Armonk, NY, USA) was used for the statistical analysis. The differences in the nerve and muscle changes of the two groups were assessed using Fisher’s exact test. To compare the expression of neurofilament and MBP between the two groups in histological staining, the intensity of neurofilament and MBP in the two groups were analyzed using an analysis of variance. A P-value of < 0.05 was considered statistically significant.

## Results

### MR findings

**Fig 3:**
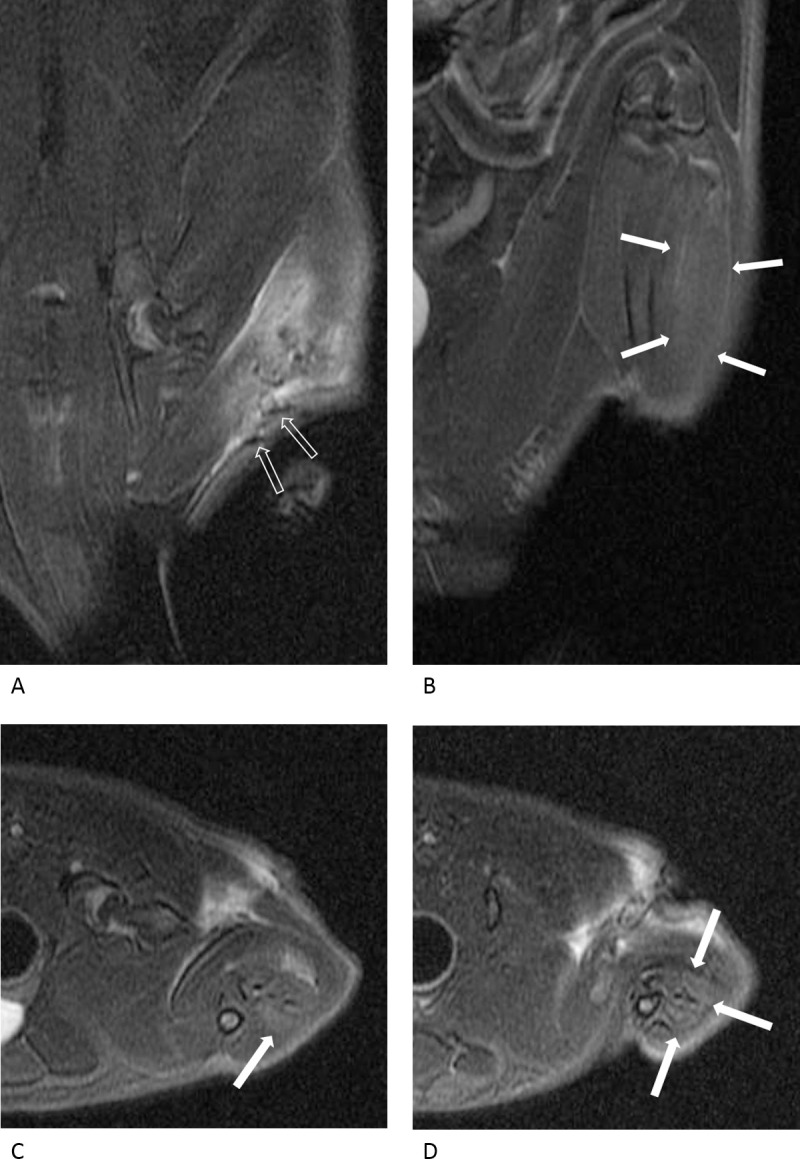
MR images of a rat with a crush injury three days after the surgery. In the oblique coronal fat-suppressed T2-weighted images (A, B), the suture and surrounding edema can be observed at the injury site (empty arrows). The injured nerve is not clearly visible, and the nerve change is considered to be grade 1. In the axial fat-suppressed T2-weighted images (C, D), the calf muscles show slight hyperintensity compared with the surrounding muscles (thick arrows), suggesting grade 2 muscle change.

**Fig 4:**
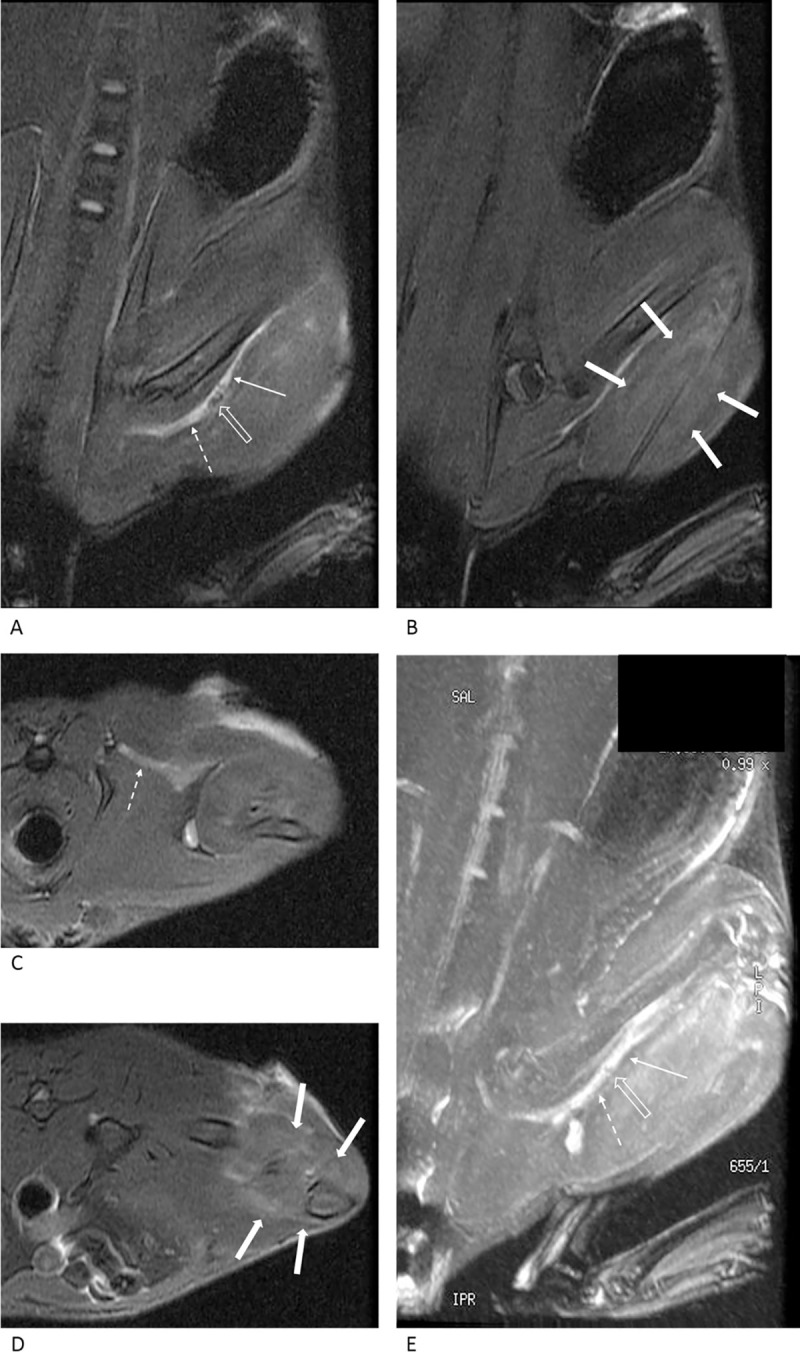
MR images of a rat with a compression injury three days after the surgery. In the oblique coronal (A, B), axial (C, D) fat-suppressed T2-weighted images and MIP image (E), the injured sciatic nerve is diffusely thickened and distinctly hyperintense. Therefore, the nerve change is classified as grade 3, which is apparent at the proximal (dashed arrows) and distal (arrows) portions of the compression site (empty arrows), revealing the suture. The calf muscles show distinct hyperintensity (thick arrows) compared with the surrounding muscles, and the muscle change is considered to be grade 3.

**Fig 5:**
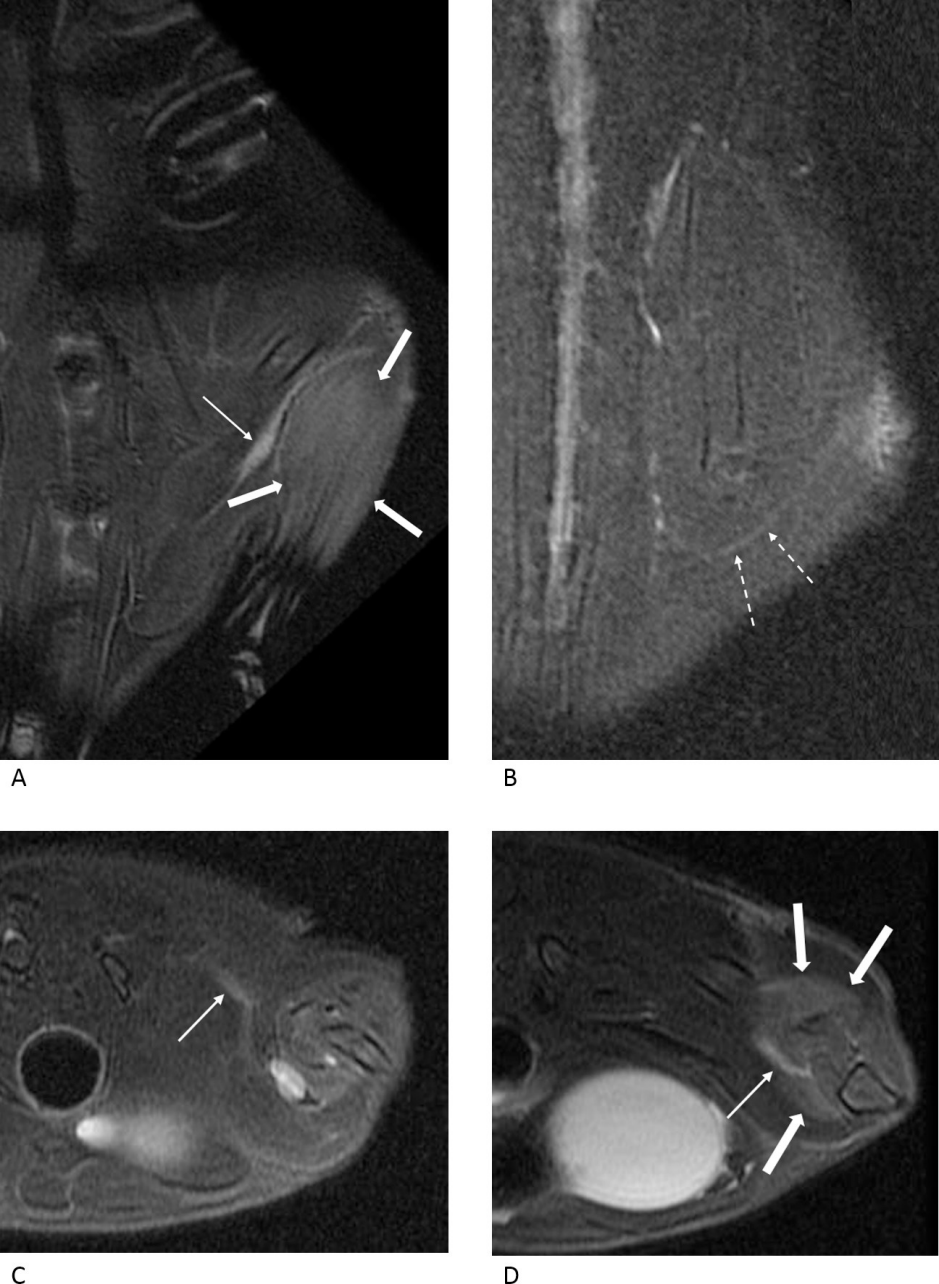
MR images of the rat with crush injury 10 days after the surgery. In the oblique coronal (A, B) and axial (C, D) fat-suppressed T2-weighted images, the injured nerve (arrows) is observed as a thick hyperintense structure, suggesting grade 3 nerve change. However, the nerve change is more prominent distal to the injury and subtle proximal (dashed arrows) to the injury. The calf muscles are distinctly hyperintense (thick arrows) compared to the surrounding muscles, suggesting grade 3 muscle change.

**Fig 6:**
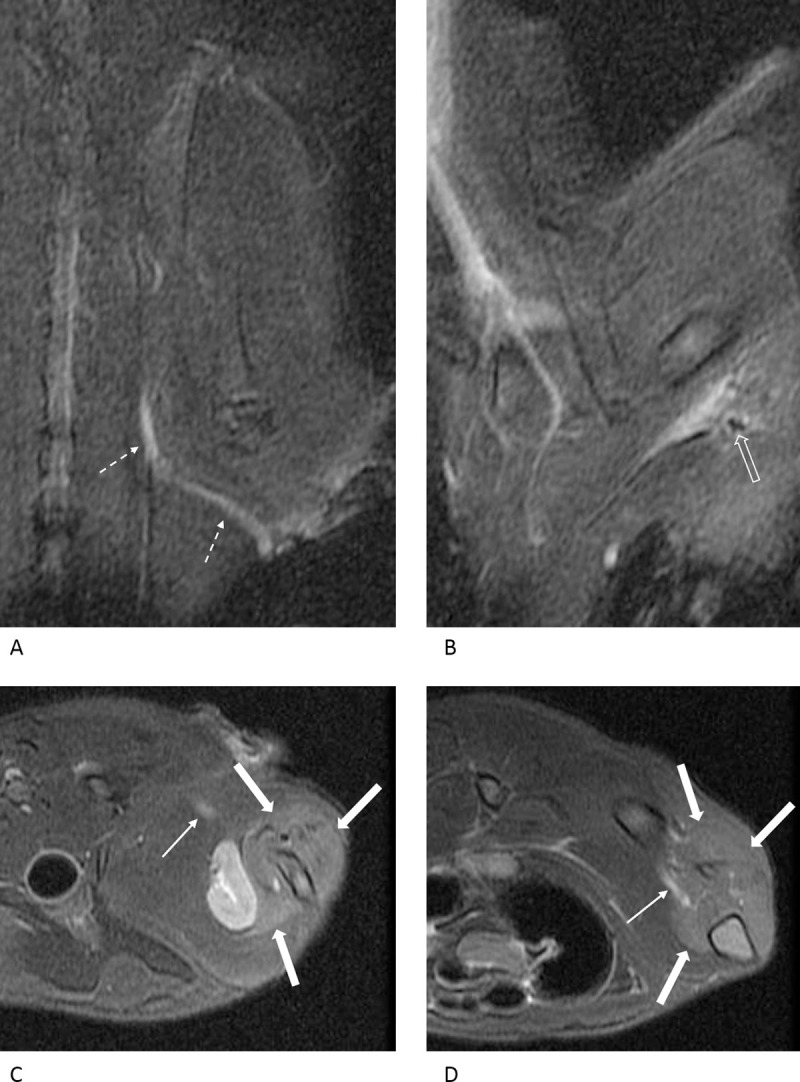
MR images of a rat with compression injury 10 days after the surgery. In the oblique coronal (A, B) and axial (C, D) fat-suppressed T2-weighted images, the injured nerve is diffusely thickened and distinctly hyperintense, suggesting grade 3 change. This change in the nerve is obvious at the proximal (dashed arrows) and distal (arrows) portions of the compression site (open arrow), showing the suture. The calf muscles show distinct hyperintensities (thick arrows) compared with the surrounding muscles, and the muscle changes are consistent with grade 3.

Nerve changes were more prominent in the compression injury group and varied significantly in the two groups on day 3 (p = 0.001) and day 10 (p = 0.026; Table 1). On day 3, four rats showed grade 1 nerve changes, and four other rats showed grade 2 nerve changes in the crush injury group (Fig 3). In the compression injury group, all rats showed grade 3 nerve changes except for one rat, which showed grade 1 nerve changes (Fig 4). On day 10, a single rat showed grade 1 changes, four rats showed grade 2 changes, and three rats showed grade 3 nerve changes in the crush injury group (Fig 5). In the compression injury group, all rats showed grade 3 nerve changes (Fig 6). Except for one rat, which showed grade 1 nerve changes on day 3 in the compression injury group, apparent edema and hyperintensity of the nerve at the proximal portion of the injury was observed in all rats with grade 3 nerve changes in the compression injury group (Figs 4 and 6). In contrast, nerve changes in the rats that showed grade 2 and 3 changes in the crush injury group were observed in the distal portion of the injury, and the changes in the proximal portion of the injury were not obvious (Figs 3 and 5).

**Table 1:** Differences in the nerve and muscle changes in the two groups.

		Grade	Crush injury group (n = 8)	Compression injury group (n = 8)	p-value
Day 3	Nerve changes	grade 1	4	1	0.001
		grade 2	4	0	
		grade 3	0	7	
	Muscle changes	grade 1	1	0	1.000
		grade 2	3	3	
		grade 3	4	5	
Day 10	Nerve changes	grade 1	1	0	0.026
		grade 2	4	0	
		grade 3	3	8	
	Muscle changes	grade 1	0	0	1.000
		grade 2	2	3	
		grade 3	6	5	

Muscle changes on days 3 and 10 were not significantly different between the two groups (p = 1.000 on both days 3 and 10). On day 3, muscle changes were observed in the crush injury group: grade 1 in one rat, grade 2 in three rats, and grade 3 in four rats. In contrast, grade 2 muscle changes were detected in three rats and grade 3 changes in five rats in the compression injury group. On day 10, grade 2 and grade 3 muscle changes were observed in two and six rats in the crush injury group, respectively, while grade 2 and grade 3 muscle changes were observed in three and five rats in the compression injury group, respectively.

### Histologic analysis

#### Histological staining

**Fig 7:**
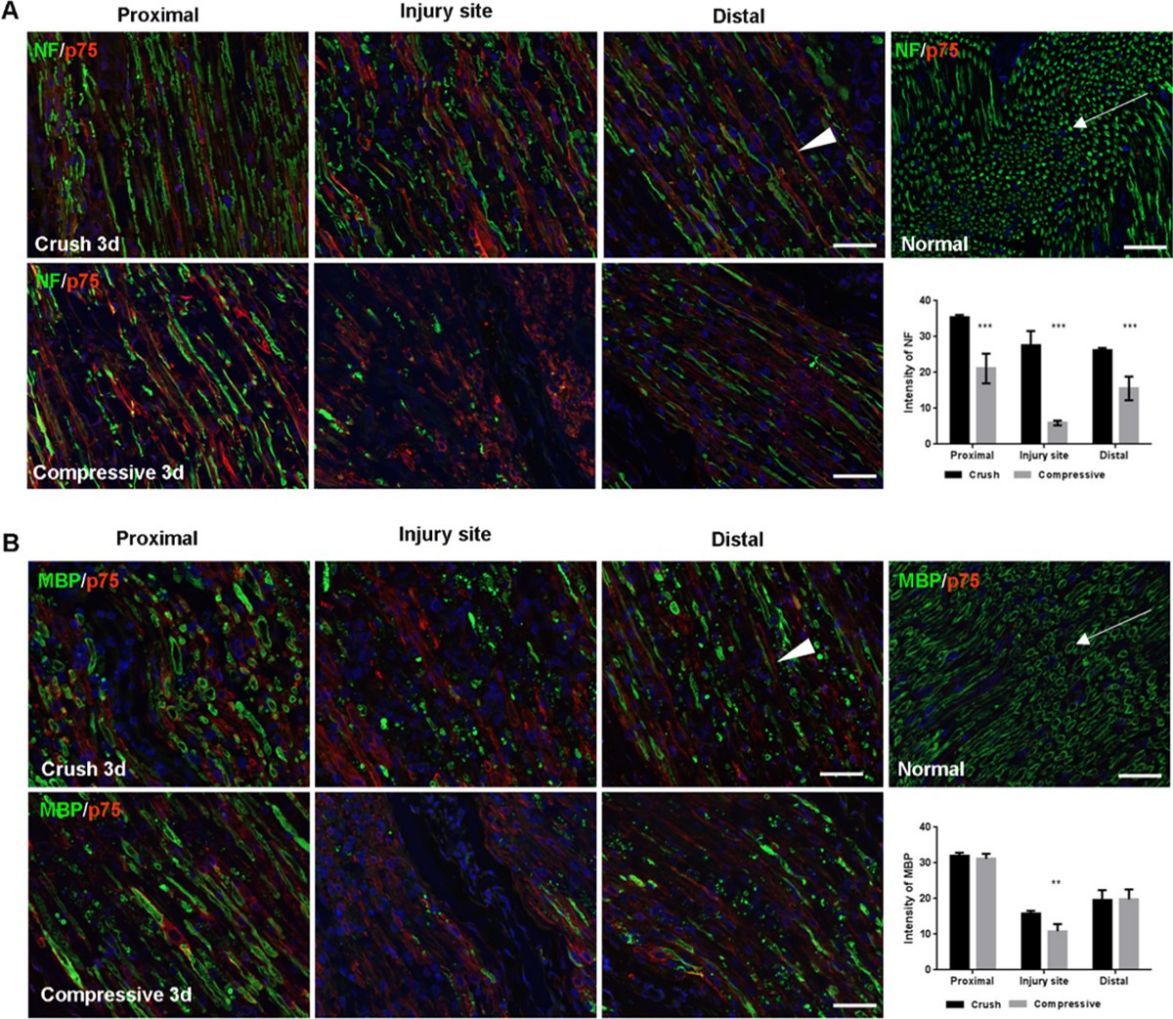
Histological staining of the nerve three days after the surgery. (A) Double staining of neurofilament (NF) and p75^NTR^ (scale bar, 50μm). Neurofilament staining shows compact dot and tubular structures in the normal peripheral nerve (arrows in the upper row, the far-right image). Neurofilament is markedly decreased in the compression injury group (lower row) compared with the crush injury group (upper row). In the compression injury group, the decrease in the levels and morphological changes of the neurofilament is pronounced proximal to the injury. Both groups stained positive for p75^NTR^ (arrowhead). Compared with the crush injury group, severe morphological changes in p75^NTR^ were detected in the compression injury group. A comparison of the intensity of neurofilament between the two groups (the rightmost graph in the lower row) showed that the amount of neurofilament expression was significantly decreased in all portions of the compression injury group compared with the crush injury group (p < 0.001). (B) Double staining of MBP and p75^NTR^ (scale bar, 50μm). MBP staining shows ring-like structures in the normal peripheral nerve (arrows in the upper row, the far-right image). MBP is markedly decreased in the compression injury group (lower row) compared with the crush injury group (upper row). In the compression injury group, the decrease in the levels and morphological changes of MBP are pronounced proximal to the injury. p75^NTR^ staining (arrowhead) shows a pattern similar to that shown in Fig 7A. A comparison of the intensity of MBP between the two groups (the rightmost graph in the lower row) shows that the amount of MBP expression at the injury site was significantly reduced in the compression injury group compared with the crush injury group (p < 0.05).

Neurofilament, the supporting structure of axons, is normally seen as a compact dot and tubular structure in histological staining (arrows in Fig 7A, green staining). In the crush injury group, neurofilament was relatively persistent in the proximal portion of the injury, but destroyed in the injury site, and decreased in the distal portion of the injury. In the compression injury group, the neurofilament level was decreased in the proximal and distal portions of the injury and was nearly absent due to severe destruction at the injury site. When comparing the intensity of neurofilament, the amount of neurofilament expression was significantly decreased in all portions of the compression injury group compared to the crush injury group (p < 0.001).

MBP, which is highly correlated with peripheral nerve myelination, normally reveals a ring-like structure under histological staining (arrows in Fig 7B, green staining). Decreased MBP levels were observed at the injury site and in the distal portion of the injury in both groups. The reduction was more pronounced in the compression injury group. When comparing the intensity of MBP, the amount of MBP expression at the injury site was significantly reduced in the compression injury group compared to the crush injury group (p < 0.05).

p75^NTR^ is not expressed in the normal peripheral nerve but is upregulated after peripheral nerve injury (arrowheads in Fig 7A and 7B, red staining). Expression of p75^NTR^ occurred in the proximal and distal portions of the injury and the injury site in both groups.

#### Western blot analysis

**Fig 8:**
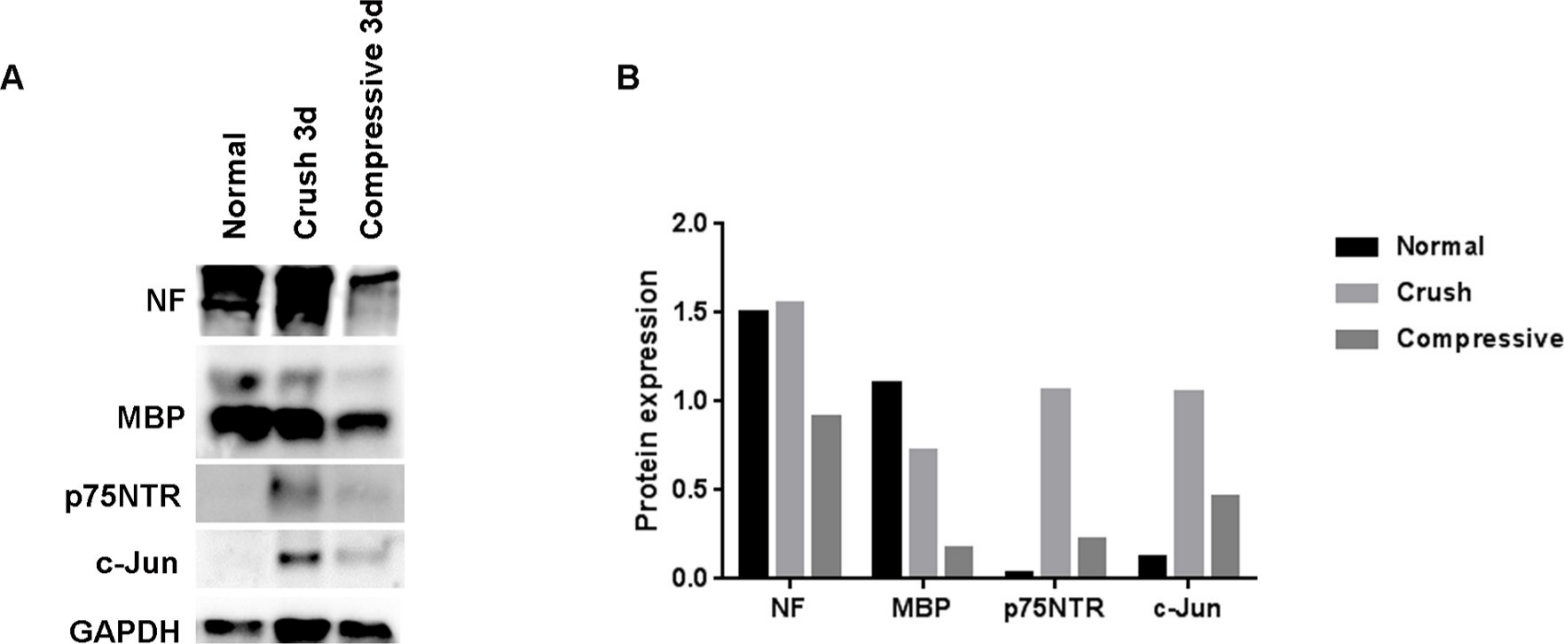
Western blot analysis of the nerve tissue at the distal portion of the injury three days after the surgery. The expression of neurofilament in the crush injury group was similar to that of the normal control group and decreased in the compression injury group. MBP expression was decreased in the compression injury group. Increased expression of p75NTR and c-jun was more pronounced in the crush injury group than in the compression injury group.

The results of the western blot analysis were similar to the histological staining results (Fig 8). The degree of neurofilament expression in the crush injury group was similar to that of the normal control group and markedly decreased in the compression injury group. MBP expression was decreased in the two groups compared with that in the normal control group, and the degree of decrease was more pronounced in the compression injury group than in the crush injury group. Expression of p75^NTR^ and c-jun was increased in both groups, and the degree of increase was more prominent in the crush injury group than in the compression injury group.

## Discussion

**Fig 9:**
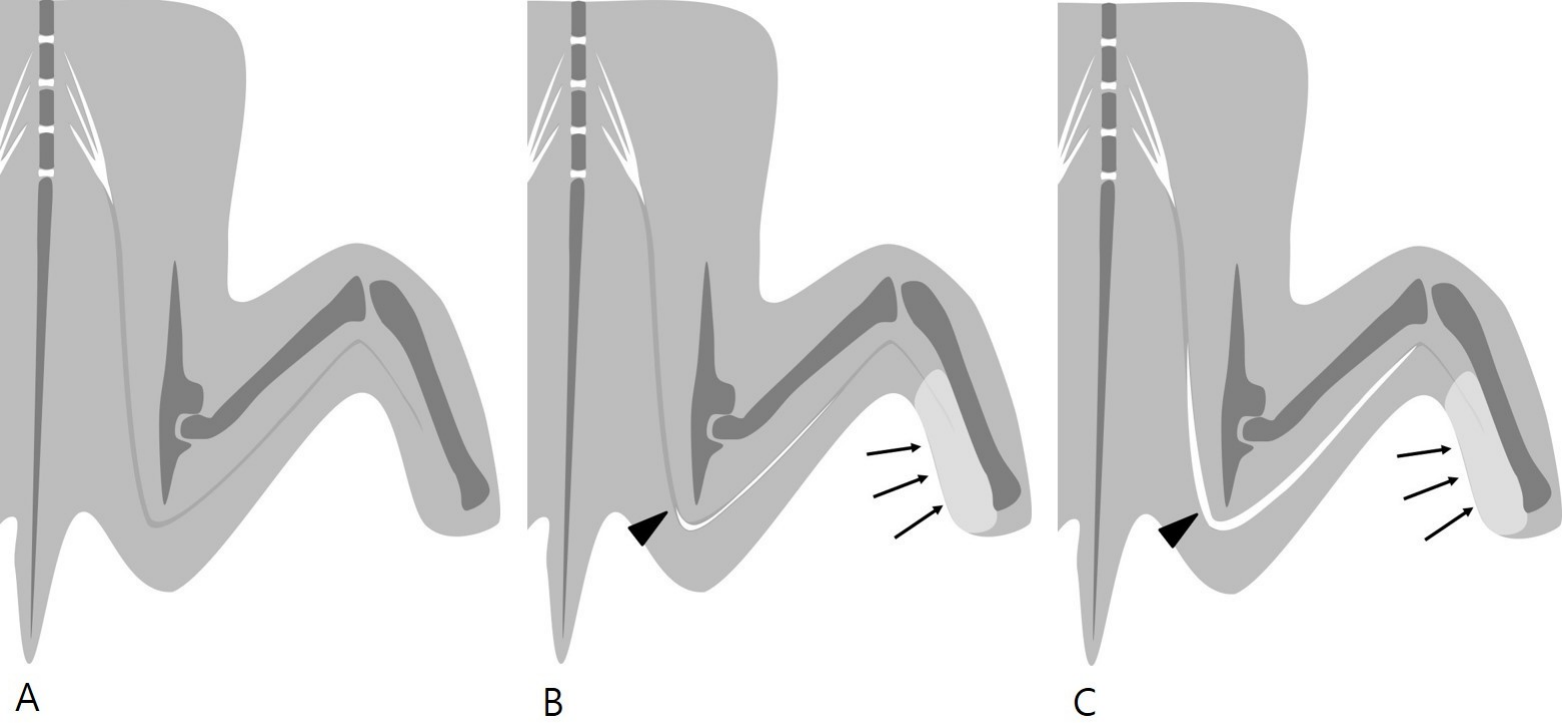
Illustration of fat-suppressed T2-weighted MR neurography in rats with normal sciatic nerve (A), acute crush injury (B), and acute compression injury (C) of the sciatic nerve. Normal sciatic nerve (A) shows intermediate to high signal intensity on fat-suppressed T2-weighted images. When an acute injury occurs, the injured nerve shows increased caliber and signal intensity. The injured nerve shows marked changes in acute compression injury (C) compared with acute crush injury (B). In acute compression injury (C), the injured nerve shows a long tubular structure, including both the proximal and distal portions of the injury. Denervation edema of the calf muscles (arrows) is similar in the two types of injury (arrowheads: injury site).

Abnormal peripheral nerves show increased caliber and high signal intensity on T2-weighted images [1–3, 6, 11–14]. In this study, the injured nerve also revealed hyperintense thickening in fat-suppressed T2-weighted images, which varied significantly between the two groups (Fig 9). The changes involving the injured nerve were more pronounced in the compression injury group. The compressed nerves were seen as long, thick, and hyperintense tubular structures. In addition, the changes were observed not only at the injury site but also distal and proximal to the injury site.

Histological staining and a western blot analysis were conducted to assess the histological changes that may be associated with different MRN findings in the two groups. Biomarkers, including neurofilament, MBP, p75^NTR^, and c-jun, were used to determine the destruction of axons and Schwann cells of the peripheral nerve. Neurofilament, the supporting structure of the axon, is a component of the mature neuronal cytoskeleton, and it decreases with axonal injury. MBP is literally “myelin basic protein” and decreases upon nerve injury. p75^NTR^, the pan-neurotrophin receptor, is not expressed in the normal nerve, but only after nerve injury. It increases during regeneration after Wallerian degeneration [20, 21]. c-jun is a transcription factor and an important protein synthesized in response to Schwann cell injury. It is expressed at a low level in the absence of nerve injury, but increases rapidly following nerve injury, and plays an important role in nerve regeneration [22].

Histopathological staining and a western blot analysis using these biomarkers showed a decrease in neurofilament levels, suggesting axonal injury, and decreased MBP, indicating significant myelin damage in the compression injury group compared with the crush injury group. Axon and myelin damage was also observed in the proximal portion of the injury in the compression injury group. These differences in the histopathological findings reflect the differences in the MRN findings between the two groups.

The western blot analysis revealed pronounced expression of p75^NTR^ and c-jun in the crush injury group than in the compression injury group, consistent with previous studies that showed an increase in the expression of p75^NTR^ and c-jun during regeneration after peripheral nerve injury [20–22]. In contrast, these two biomarkers were minimally expressed in the compression injury group. We inferred that regeneration occurred after nerve injury in the crush injury group, whereas no regeneration of peripheral nerve injury occurred in the compression injury group.

It is well known that Wallerian degeneration progresses from the injury site to the distal portion upon peripheral nerve injury [17, 23]. In this study, nerve changes were detected at the injury site and distal to the injury site. In the compression group, however, remarkable histopathological changes were found proximal and distal to the injury. Similarly, several animal studies of the sciatic nerve showed T2 hyperintensity proximal to the injury [16, 24, 25]. The authors speculated that this might be attributed to the interruption of axoplasmic flow, increasing axoplasm proximal and distal to the injury site, venous obstruction increasing endoneurial fluid, and changes in bulk flow resulting in extended signal variation along the length of the nerve [24]. In a study on traction injury in rabbits, the proximal portion of the injured nerve, as well as the injury site and the distal portion, were enlarged and hyperintense on T2-weighted images, which was attributed to “retrograde degeneration” [25]. In this study, we supposed that retrograde degeneration occurred markedly in the compression injury group.

This suggests that increased signal intensity of the abnormal nerve on T2-weighted images results from vascular congestion, decreased axoplasmic flow causing abnormal accumulation of endoneurial fluid in the proximal portion, and Wallerian degeneration in the distal portion [1, 11]. In this study, axonal and myelin damage in the histopathological study occurred in the region of the injured nerve, which was analyzed with MRN.

Recently, various MR imaging techniques, including diffusion-weighted imaging, diffusion tensor imaging (DTI), and magnetization transfer ratio (MTR) imaging, have been used to evaluate peripheral neuropathy and its recovery [26–28]. Giorgetti et al. demonstrated that MTR is a feasible modality for the evaluation of peripheral nerve injury in mouse models [27]. They suggested that decreased MTR reflects decreased myelin concentration from age-related changes as well as traumatic injury. In the study by Dortch et al., the authors showed the clinical application of MTR in patients with Charcot-Marie-Tooth disease [26]. Thus, various MR imaging techniques may reflect the histological changes of peripheral neuropathy in patients as well as experimental animals. Moreover, MTR and DTI can be used to evaluate the therapeutic effect of the injured nerve and its regeneration [27, 28], which is beyond the scope of this study.

The MRN findings differed between the two groups, similar to the histopathologic study. Thus, MRN might be used in the differential diagnosis of acute traumatic peripheral nerve injury and acute compressive neuropathy.

The most significant limitation of this study is that the crush injury model may not represent traumatic peripheral nerve injury triggered by various factors. Several studies have reported MRI findings of the injured nerve with varying severity and types [14, 16–18, 23]. In these studies, T2 hyperintensity and contrast enhancement of the nerve was more prominently associated with severe injury. Crush injury in this study corresponded to axonotmesis [16]. Because early recovery is expected in milder injury-causing neurapraxia, we did not include this type of injury in this study due to its limited clinical significance. Neurotmesis was not included as a neuroma as it is frequently visible on MRI. Furthermore, as crush injury has been widely investigated in other studies, it might be reasonable to compare the crush injury model with the compressive neuropathy model.

In this study, we hypothesized that the severity (strength), type, and duration of the injury might result in different MRN and histopathological findings. As differences in the severity of the injury may induce different changes, we exposed rats to a similar force. In the crush injury group, the nerve was directly crushed using forceps for 5 s. In the compression injury group, we induced a similar degree of injury by ligating the nerve, resulting in obvious compression. We interpreted that the most significant difference between the two groups was the duration of nerve injury. However, the difference in the severity of the injury could not be objectively evaluated. In the crush injury group, nerve injury was applied for 5 s, and in the compression injury group, the duration of the injury was longer, as the nerve was ligated for three to 10 days until the MR scan was performed. Therefore, we suggest that degeneration was followed by regeneration in the crush injury group, and regeneration failed under prolonged axon and myelin damage in the compression injury group. In brief, different injury mechanisms based on the differences in the duration of injury may result in different MRN findings in the two groups.

There were several other limitations to the current study. First, the small sample size resulted in limited statistical power. Histopathological studies were conducted in only four rats. The main purpose of this study was to elucidate the differences in the MRN findings between the two groups. To identify direct and objective relationships between the MRN findings and histopathological changes, further histopathological studies are required. Second, the histopathologic study was conducted only on day 3 after surgery. Differences in the MRN findings between the two groups were statistically significant on both day 3 (p = 0.001) and day 10 (p = 0.026) but were more pronounced on day 3. Therefore, a histopathologic study was conducted on day 3. Additional studies, including a histopathological analysis on day 10, are necessary to correlate MRN findings with histopathological changes more clearly. Third, MR images were qualitatively analyzed using the means of the scores. The rat sciatic nerve was small and not evenly visualized throughout the length. For this reason, a quantitative analysis, including MR signal intensity and nerve thickness, could not be applied. Fourth, functional assessments such as a nerve conduction study and walking track analysis were not conducted in this study. Further studies, including these functional analyses, would be useful. Fifth, compression injury that causes entrapment syndrome is frequently encountered in daily practice. Entrapment syndrome may be a chronic compression injury induced by loose ligation where the nerve is not grossly compressed. This type of injury may show completely different MRN findings. Therefore, further studies are necessary. Finally, additional studies are needed to apply the results of this study to human peripheral neuropathy encountered in clinical practice. In the human body, variables such as the caliber of the involved nerve and precise anatomical localization, as well as the degree of injury, may alter the MRN findings. Despite these limitations, this study compared the MRN findings of peripheral nerve injury using relatively common and reproducible peripheral nerve injury models.

In conclusion, the injured nerve showed distinct changes in the long segment in the acute compression nerve injury model compared with the acute traumatic nerve injury model on MRN, which were closely associated with the histopathological changes. Therefore, MRN is expected to be a useful modality in the differential diagnosis of acute traumatic peripheral nerve injury and acute compressive neuropathy.

## Supporting information

S1 File The original images of western blotting at the distal portion of the injury three days after the surgery. (PDF)
