# Supplementary figures and images for: Comparison of MR findings of acute traumatic peripheral nerve injury and acute compressive neuropathy in a rat model
Source: PLoS One. 2020 Nov 19;15(11):e0240911. doi: 10.1371/journal.pone.0240911 (PMC7676645; doi:10.1371/journal.pone.0240911)

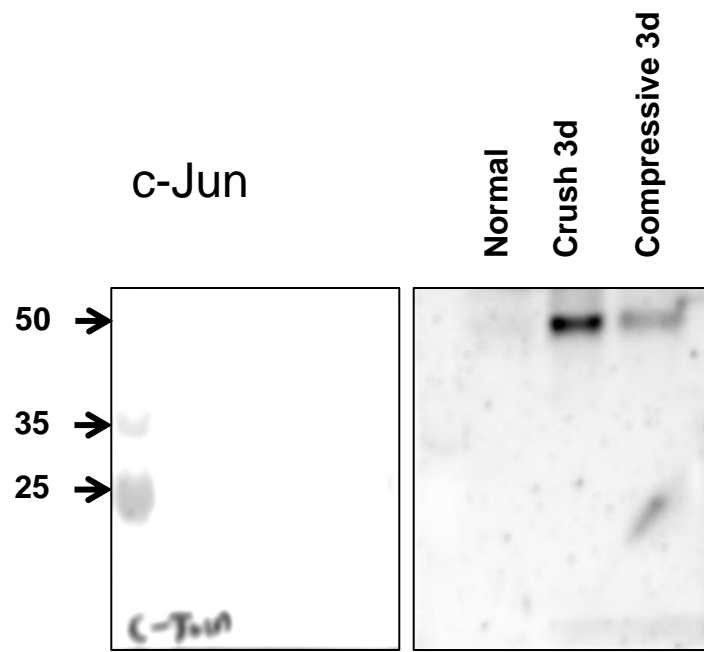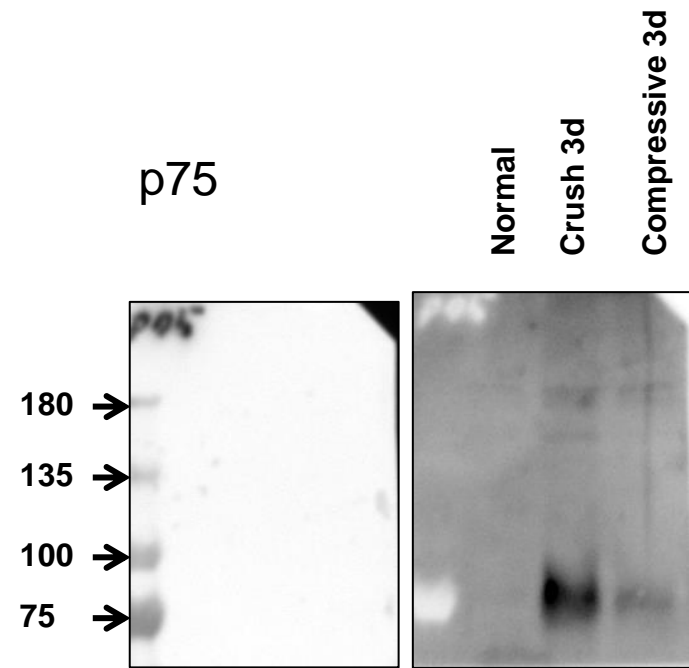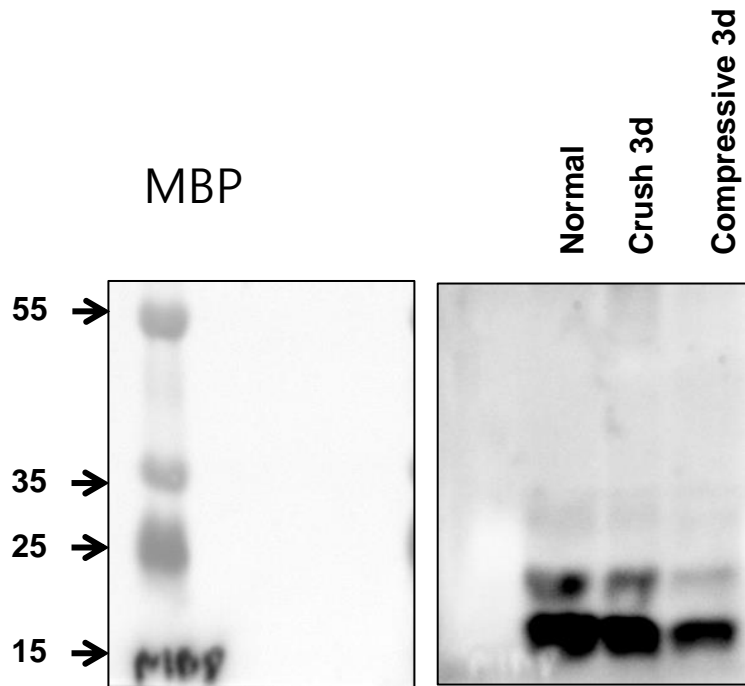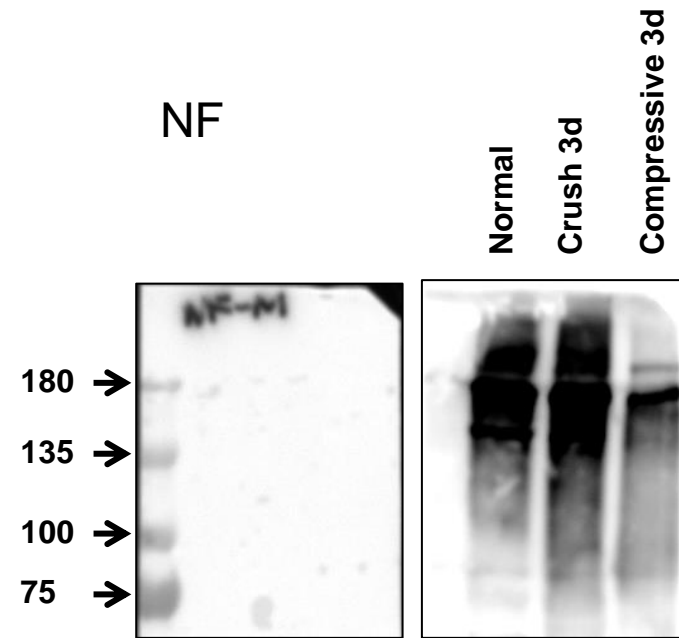

GAPDH

35 →

25 →

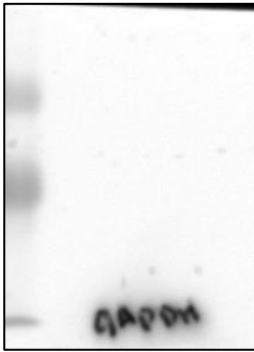

Normal

Crush 3d

Compressive 3d

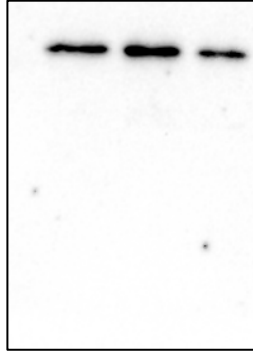

Supplement: S1 File — (PDF) [file pone.0240911.s001.pdf]
